# Unveiling the Ability of Witch Hazel (*Hamamelis virginiana* L.) Bark Extract to Impair Keratinocyte Inflammatory Cascade Typical of Atopic Eczema

**DOI:** 10.3390/ijms23169279

**Published:** 2022-08-17

**Authors:** Stefano Piazza, Giulia Martinelli, Andrea Magnavacca, Marco Fumagalli, Carola Pozzoli, Massimo Terno, Luisa Canilli, Marco Angarano, Nicole Maranta, Mario Dell’Agli, Enrico Sangiovanni

**Affiliations:** 1 Department of Pharmacological and Biomolecular Sciences, University of Milan, 20133 Milan, Italy; 2 Ganassini Institute S.p.A. (Istituto Ganassini), 20139 Milan, Italy

**Keywords:** *Hamamelis virginiana*, witch hazel, phytotherapy, atopic dermatitis, eczema, allergy

## Abstract

*Hamamelis virginiana* L. bark extract is a traditional remedy for skin affections, including atopic dermatitis/eczema (AD). Hamamelis preparations contain tannins, including hamamelitannin (HT), although their pharmacological role in AD is still unknown. This study aimed to study the rational for its topical use by considering the impact of crucial biomarkers on AD pathogenesis. A standardized extract (HVE) (0.5–125 μg/mL) was compared to hamamelitannin (HT), its main compound (0.5–5 μg/mL), in a model of human keratinocytes (HaCaTs), challenged with an AD-like cytokine milieu (TNF-α, IFN-γ, and IL-4). HVE inhibited the release of mediators involved in skin autoimmunity (IL-6 and IL-17C) and allergy (TSLP, IL-6, CCL26, and MMP-9) with a concentration-dependent fashion (IC_50s_ < 25 μg/mL). The biological mechanism was ascribed, at least in part, to the impairment of the NF-κB-driven transcription. Moreover, HVE counteracted the proliferative effects of IL-4 and recovered K10, a marker of skin differentiation. Notably, HT showed activity on well-known targets of IL-4 pathway (CCL26, K10, cell proliferation). To the best of our knowledge, this work represents the first demonstration of the potential role of *Hamamelis virginiana* in the control of AD symptoms, such as itch and skin barrier impairment, supporting the relevance of the whole phytocomplex.

## 1. Introduction

*Hamamelis virginiana* L. has long-standing use in Europe and USA for the treatment of varicose veins, hemorrhoids, and minor skin disorders [1,2]. The medicinal use regarding topical preparations is based on distillate or polar extracts from bark and twigs (Hamamelidis aqua and Hamamelidis cortex, respectively) [2]. Several clinical trials on patients with eczema and solar erythema were conducted using distillates with conflicting results [3,4,5]; on the contrary, polar extracts were poorly investigated, although they showed a general anti-inflammatory activity in several in vivo studies [6,7]. Tannins are the main compounds reported in polar extracts, while they are theoretically absent in distillates, which contain mainly volatile compounds [2]. Among tannins, the most abundant is hamamelitannin (HT) (M.W: 484.4 mg/mol), but proanthocyanidins (condensed tannins) have also been reported [8,9]. The bioactivity of tannins-enriched fractions or isolated tannins was evaluated by in vitro studies, in which potential antibacterial, antioxidant, and vasoprotective properties were suggested for HT, while antiviral and anti-inflammatory properties were proposed for proanthocyanidins [9,10,11,12]. Despite the broad use of *Hamamelis virginiana* in topical preparations, studies involving keratinocytes are rare. We recently published an in vitro study, suggesting for the first time that a tannin-rich extract from *Hamamelis virginiana* may contribute to counteract skin inflammation during *C. acnes* infection; however, the contribution of HT was excluded [13].

Atopic dermatitis (AD) is one of the most prevalent chronic diseases in the world, devoid of a definitive cure, with an increasing incidence in children, similarly to other allergic disorders [14]. The skin of AD patients is characterized by dryness, thickness, redness, and itch, associated with lesions and increased risk of bacterial infection. The recent discoveries regarding the pathogenesis of atopic dermatitis (AD) renewed the research for novel therapeutic strategies based on immunomodulation [15]. Keratinocytes were recognized as playing a crucial role in bridging the environmental insults and the host response, mainly, but not exclusively, orchestrated by Th2 lymphocytes (for an extensive review on the topic, see [16]). In fact, Th2 cytokines and environmental factors, such as physical damage and microbes, can promote the release of TSLP from keratinocytes, thus triggering the sensitization process, involving dendritic cells, B cells, mast cells, and eosinophils [17]. The typical phenotype of AD is characterized by elevated levels of interleukins belonging to type 2 inflammation (IL-4, IL-5, IL-6, and IL-13) at the keratinocyte level, which downregulate markers of epidermal differentiation and barrier homeostasis, such as K10, involucrin, and filaggrin [18,19,20], and promote the release of pro-allergic chemokines, such as eotaxins (CCL11, CCL24, and CCL26) [21].

In addition, innate immunity cytokines (IL-1β, TNF-α) and Th1/Th17 mediators (IFN-γ, IL-12, and IL-17) were implicated in the skin damage occurring in AD patients [22]. Of note, the combination of TNF-α (representative of innate response) with IFN-γ or IL-4 (representative of the Th1 and Th2 response, respectively) was successfully used to mimic the inflammatory environment of AD in keratinocytes [23,24,25]. Accordingly, NF-κB, a well-known master regulator of TNF-α-inducible genes, is a recognized pharmacological target in both type 1 and type 2 inflammation [26].

Polyphenol-rich plants are widely used in cosmetics with hydrating and soothing properties, but we also recently underlined their potential pharmacological activities at the skin level, such as antioxidant, anti-allergic, and anti-inflammatory activities [27]. Among polyphenols, flavones (e.g., apigenin) from plant extracts have a long medicinal tradition against allergic disorders, recently sustained by a body of in vivo evidence [28,29]. On the contrary, tannin-containing plants such as *Hamamelis virginiana* L. were scarcely investigated in models of AD. Based on this background, the present work aimed to validate the role of a polar extract from *Hamamelis virginiana* L. and pure HT for the first time in a model of keratinocytes (HaCaTs), challenged with cytokines mimicking the AD phenotype.

## 2. Results

### 2.1. HVE, but Not HT, Inhibits IL-17C and MMP-9 Release in HaCaT Cells

**Figure 1:**
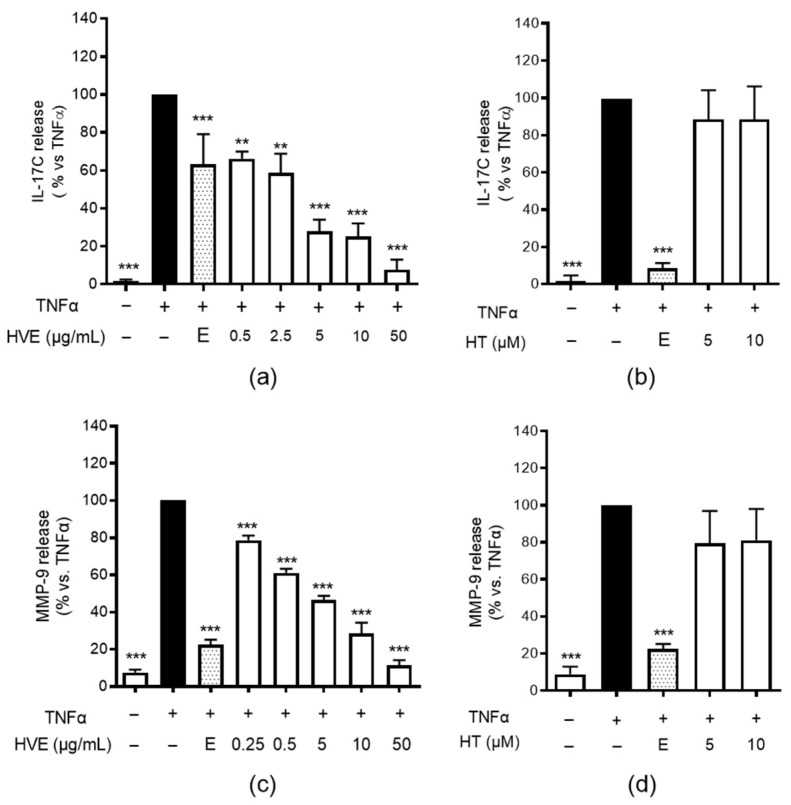
The effect of HVE and HT treatment (24 h) on the release of IL-17C (**a**,**b**) and MMP-9 (**c**,**d**) by HaCaT cells challenged by TNF-α (10 ng/mL), measured by the ELISA assay. HT was not effective until the highest concentration tested (10 μM corresponding to 4.93 μg/mL). The data are expressed in percentages, relative to the stimulated control, which is arbitrarily assigned a value of 100%. ** *p* < 0.01, *** *p* < 0.001 versus stimulus. HVE, *Hamamelis virginiana* bark extract; reference inhibitor: E, epigallocatechin gallate (20 μM).

In our previous research on the anti-inflammatory activity of HVE in HaCaT cells, we observed the promising inhibitory effects of hamamelis glycolic extract on several TNF-α-induced mediators [13]. Cytokines belonging to the IL-17 family are known to regulate epithelial barrier homeostasis during infections, but also to contribute in autoimmune processes, in which TNF-α plays a crucial role (for a review on the topic, refer to Pappu et al. [30]). The expression of IL-17C is increased by TNF-α in keratinocytes and has been suggested as a epithelial biomarker in the onset of skin-inflammatory diseases, including psoriasis and AD [31,32]. HVE was able to impair IL-17C release with a concentration-dependent fashion (IC_50_ = 1.53 μg/mL) (Figure 1a). In line with our previous paper, HT was unable to exert an inhibitory effect (Figure 1b), thus suggesting the main contribution of condensed tannins, the second most abundant class of compounds.

Other mediators known to influence skin homeostasis during the inflammatory process are matrix metalloproteases (MMPs). MMPs, such as MMP-9, are implicated in several allergic diseases including AD, in which they may alter tissue repair in favor of skin rearrangement [33]. Consequently, we investigated the effects of HVE on MMP-9 release induced by TNF-α. According to the previous results, HVE (Figure 1c), but not pure HT (Figure 1d), inhibited MMP-9 release with similar IC_50_ (1.11 μg/mL) relating to IL-17C.

### 2.2. HVE and HT Inhibit TSLP Release in HaCaT Cells

TSLP is mainly responsible for the sensitization process of dendritic cells occurring in AD and also directly promotes skin barrier impairment [16,17] and neurogenic pruritus [34]. As mentioned in the introduction, the recognized triggers of TSLP production are microbial structures and Th2 cytokines, among which IL-4 has received the most attention as a clinical target in AD. Of note, monoclonal antibodies against both tezepelumab (TSLP) and dupilumab (IL-4) have been successfully assessed in clinical trials [35].

The most relevant source of TSLP are damaged keratinocytes from the outer layers of the epidermis [36]. Despite the fact that the machinery for TSLP expression is not completely clear, several authors assigned a crucial role to the transcription factors STAT6 and NF-κB [37,38], i.e., well-known signal transducers downstream of IL-4R and TNFR, respectively. In line with Kim et al. [24], we observed that differentiated keratinocytes can release TSLP after the stimulation with the combination of TNF-α and IL-4, but not by the stimuli taken individually. Moreover, we confirmed that IFN-γ was unable to enhance its release (data not shown), thus supporting the idea that STAT6 activation is required to enhance the TNF-α effect. On these bases, the TNF-α/IL-4 combination was considered a pro-allergic challenge.

**Figure 2:**
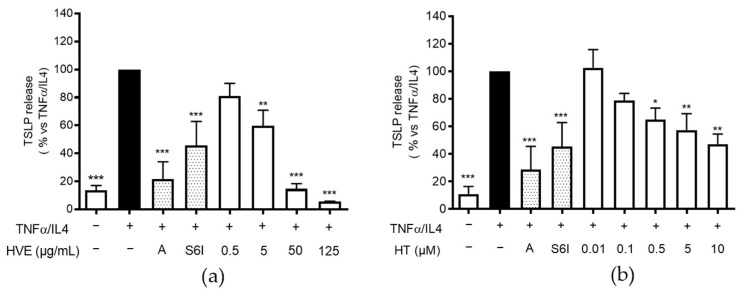
The inhibitory effect of HVE (**a**) and HT (**b**) treatment (24 h) on the release of TSLP using differentiated HaCaT cells challenged by TNF-α (10 ng/mL)/IL-4 (100 ng/mL), measured by the ELISA assay. The highest concentration of HT (10 μM) corresponds to 4.93 μg/mL. The data are expressed in percentages, relative to the stimulated control, which is arbitrarily assigned a value of 100%. * *p* < 0.05, ** *p* < 0.01, *** *p* < 0.001 versus stimulus. HVE, *Hamamelis virginiana* bark extract; HT, hamamelitannin; reference inhibitors: A, apigenin (20 μM); S6I, STAT-6 inhibitor (50 nM).

Consequently, in the following experiments, we included a known anti-allergic and anti-inflammatory polyphenol (apigenin 20 μM, A) [28,29] and the selective STAT6 inhibitor AS1517499 (S6I, 50 nM), in comparison with HVE and HT. In this context, HVE impaired the release of TSLP with a concentration-dependent fashion, with an IC_50_ of 4.33 μg/mL (Figure 2a). Surprisingly, HT exerted the same biological activity (IC_50_ = 2.56 μM), although at concentrations higher than those sufficient for HVE (Figure 2b). In fact, the highest concentration tested for HVE (125 μg/mL) only parallels a moderate inhibitory effect for the corresponding HT 0.3% (0.74 μg/mL or 1.5 μM), thus suggesting a partial contribution or a possible synergistic effect among polyphenols. Of note, both apigenin and S6I showed a significant inhibitory effect.

This observation represents the first demonstration of an inhibitory activity exerted by isolated HT in our experimental model, thus raising further questions around the biological properties of HT and HVE.

### 2.3. HVE, but Not HT, Inhibits IL-6 Release in HaCaT Cells

IL-6 is known to promote Th2 polarization and B cell proliferation, but it also plays a direct role in skin barrier impairment and inflammation in chronic AD [39,40,41]. In our experimental settings, TNF-α was only slightly effective in enhancing IL-6 production by HaCaT cells. Despite IL-6 is known to be NF-κB activation-dependent, other authors demonstrated that IFN-γ or IL-4 may enhance IL-6 release in keratinocytes through STAT1/STAT6 signaling [42,43]. Accordingly, we observed that the addition of these cytokines to TNF-α caused a strong increase in IL-6 release after 24 h of treatment; on the contrary, IL-4 and IFN-γ were ineffective when used individually.

**Figure 3:**
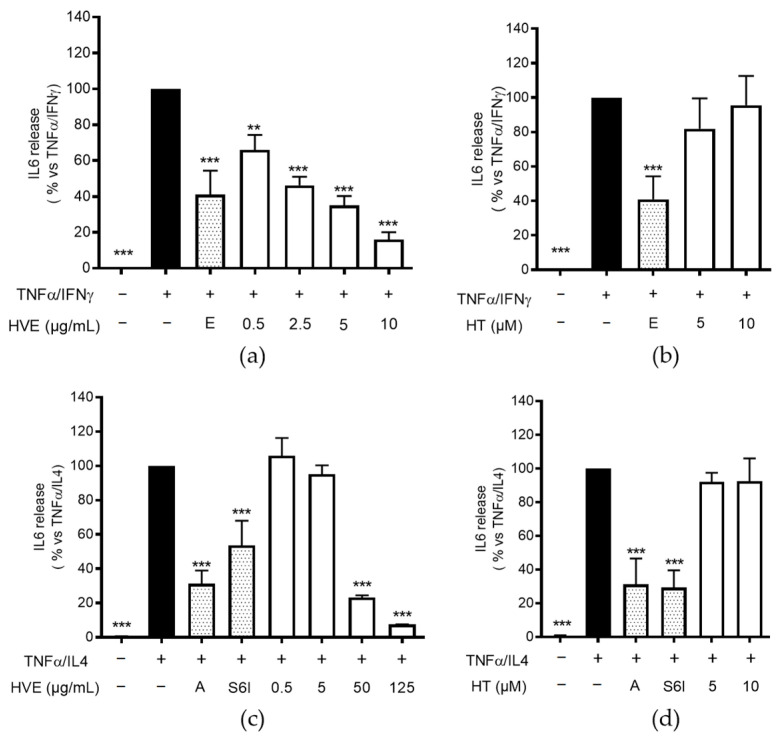
The effect of HVE and HT treatment (24 h) on the release of IL-6 using differentiated HaCaT cells challenged by TNF-α (10 ng/mL)/IFN-γ (10 ng/mL) (**a**,**b**) or TNF-α (10 ng/mL)/IL-4 (100 ng/mL) (**c**,**d**), measured by the ELISA assay. HT was not effective until the highest concentration tested (10 μM corresponding to 4.93 μg/mL). The data are expressed in percentages, relative to the stimulated control, which is arbitrarily assigned a value of 100%. ** *p* < 0.01, *** *p* < 0.001 versus stimulus. HVE, *Hamamelis virginiana* bark extract; reference inhibitors: E, epigallocatechin gallate (20 μM); A, apigenin (20 μM); S6I, STAT-6 inhibitor (50 nM).

Consequently, HVE and HT were evaluated against TNF-α/IFN-γ or TNF-α/IL-4 combinations. Again, HVE exerted a concentration-dependent inhibitory effect on IL-6 release, with an IC_50_ of 2.70 μg/mL, while HT was not active against TNF-α/IFN-γ challenge (Figure 3a,b). In line with the previous results regarding TSLP release, HVE also inhibited IL-6 release against TNF-α/IL-4 challenge (IC_50_ = 21.30 μg/mL) (Figure 3c), but contrarily to what expected, HT was not active at the concentrations tested (1–10 μM) (Figure 3d).

Of note, our setting showed only a partial overlap between IL-6 and TSLP stimulatory conditions. In particular, IFN-γ played a role only in IL-6 release, thus reflecting a different mechanism for IL-6 induction. On the other hand, the inhibitory effects of S6I 50 nM on both TSLP and IL-6 release suggested that HT may have counteracted TSLP release in a STAT6-independent way.

### 2.4. HVE, but Not HT, Inhibits the NF-κB-Driven Transcription

We already demonstrated the biological activity of HVE against TNF-α-induced proinflammatory mediators, including NF-κB [44]. In the same context, the involvement of HT in the impairment of NF-κB pathway was excluded. For this reason, we wondered whether HVE might be able to counteract TSLP and IL-6 release through the same mechanism. As briefly mentioned, NF-κB is a crucial transcription factor for the expression of all the cytokines investigated until this point of the study. Given the importance of TNF-α co-stimulatory cytokines in the context of the present work, we investigated if HVE may impair the NF-κB pathway even after the additional stimulation with IFN-γ or IL-4. Moreover, to elucidate the peculiar inhibitory effects of HT on TSLP release, the compound was compared with HVE against TNF-α/IL-4 challenge.

**Figure 4:**
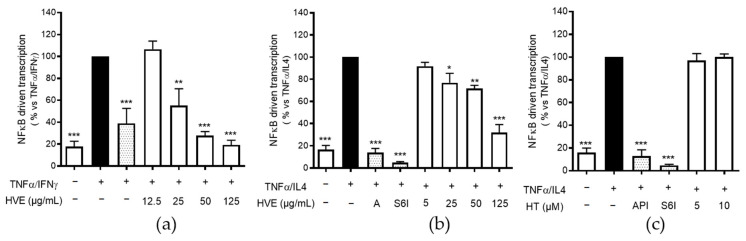
The inhibitory effect of HVE treatment (24 h) on the NF-κB-driven transcription in HaCaT cells challenged by TNF-α (10 ng/mL)/IFN-γ (10 ng/mL) (**a**) or TNF-α (10 ng/mL)/IL-4 (100 ng/mL) (**b**), measured by plasmid transfection. The involvement of HT (10 μM corresponding to 4.93 μg/mL) in the inhibitory effect against TNF-α/IL-4 challenge was excluded (**c**). The data are expressed in percentages, relative to the stimulated control, which is arbitrarily assigned a value of 100%. * *p* < 0.05, ** *p* < 0.01, *** *p* < 0.001 versus stimulus. HVE, *Hamamelis virginiana* bark extract; reference inhibitors: A, apigenin (20 μM); S6I, STAT-6 inhibitor (50 nM).

As expected, HVE inhibited the transcriptional activity of NF-κB during both TNF-α/IFN-γ or TNF-α/IL4 challenges, with IC_50_s of 23.20 and 55.50 μg/mL, respectively (Figure 4a,b), while the involvement of HT was once again excluded up to the highest concentration tested of 10 μM (Figure 4c). Surprisingly, S6I (50 nM) was able to completely block the NF-κB-driven transcription, thus suggesting a strong involvement of STAT6 interaction at an upstream level during IL-4 co-stimulation.

These data supported the idea that HVE may exert its biological activity mainly through the NF-κB impairment, regardless of the possible interference with the IL-4 pathway. On the contrary, these data allowed us to exclude the involvement of STAT6 and NF-κB in the bioactivity of HT, thus opening up speculation surrounding the selective effects on the IL-4 pathway. Consequently, to better characterize the bioactivity of HVE and HT, we further investigated several IL-4-dependent targets with a relevant role in AD.

### 2.5. HVE and HT Inhibit IL-4-Induced CCL26 in HaCaT Cells

The previous results suggested that HT may contribute to TSLP inhibition through mechanisms unrelated with STAT6 and NF-κB pathways. Therefore, we supposed that HT may contribute to the bioactivity of HVE acting at different levels of the IL-4 pathway, selectively. For example, IL-4 is known to induce skin barrier impairment also through the activation of several MAPKs in keratinocytes [45]. To sustain our hypothesis, we evaluated the effects of HVE and HT on relevant outcomes of IL-4 stimulation in keratinocytes, in accordance with other authors [46,47], including the release of CCL26 (eotaxin-3), the impairment of skin barrier, and the induction of proliferation.

**Figure 5:**
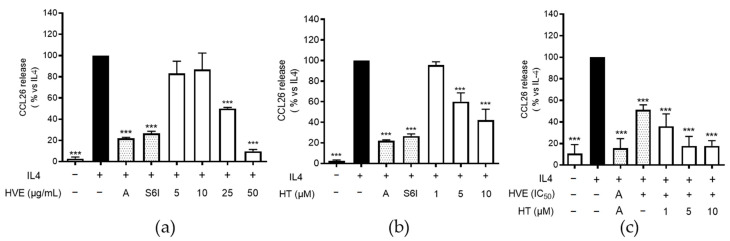
The inhibitory effect of HVE (**a**) and HT (**b**) treatment (24 h) on the release of CCL26 using differentiated HaCaT cells challenged by IL-4 (100 ng/mL), measured by the ELISA assay. The effect of HT (10 μM corresponding to 4.93 μg/mL) on HVE IC_50_ (20 μg/mL) is also reported (**c**). The data are expressed in percentages, relative to the stimulated control, which is arbitrarily assigned a value of 100%. *** *p* < 0.001 versus stimulus. HVE, *Hamamelis virginiana* bark extract; HT, hamamelitannin; reference inhibitors: A, apigenin (20 μM); S6I, STAT-6 inhibitor (50 nM).

CCL26 is a crucial chemokine involved in the recruitment of eosinophils during AD and was reported as one of the most upregulated genes in IL-4-challenged HaCaT cells [47,48]. As reported in Figure 5a, HVE inhibited CCL26 release with an IC_50_ of 21.36 μg/mL. As supposed, HT resembled the inhibitory activity of HVE, although at concentrations 100-fold higher than those present in the extract (Figure 5b). However, to clarify the role of HT for the phytocomplex, we added increasing concentrations of HT (1, 5, and 10 μM) to a concentration of HVE close to the IC_50_ (20 μg/mL), thus revealing an additional inhibitory effect already at HT 1 μM (Figure 5c). These data might suggest a synergistic effect among HVE polyphenols and sustain the importance of the whole phytocomplex.

### 2.6. HVE and HT Inhibit IL4-Induced Proliferation and Recover K10 Expression

IL-4 is involved in the abnormal proliferation of different cell types beyond T cells, such as vascular and epithelial cells [49,50]. It exerts a pleiotropic effect also on keratinocytes, causing disturbance in the proliferation–differentiation balance, thus promoting skin thickness and compromising wound healing and skin barrier [20,46]. The full terminal differentiation of corneocytes requires the multilayered skin structure; nevertheless, as mentioned in our previous paper [44], we observed the increase in K10 expression during the cultivation of HaCaT cells in a high-calcium medium, which correlated with a limited increase in IVN after 9 days. K10 and IVN were selected as documented targets of IL-4 in AD pathogenesis, and well-known biomarkers of early and terminal differentiation, respectively [19,45]. 

**Figure 6:**
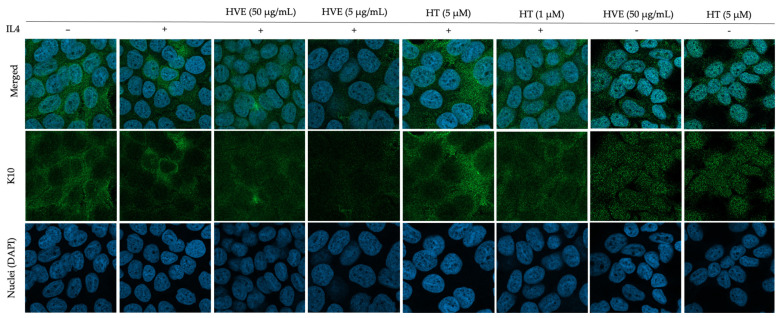
Immunofluorescence images reporting the effect of HVE and HT treatment (24 h) on the impairment of K10 expression induced by IL-4 (100 ng/mL) in differentiated HaCaT cells. HVE, *Hamamelis virginiana* bark extract; HT, hamamelitannin. Green, K10 staining; blue, DAPI (nuclei) staining.

In line with the literature, the immunofluorescence experiments confirmed that IL-4 was sufficient to impair K10 expression; consequently, we evaluated the potential role of HVE and HT on this target. Once again, we observed that HVE and HT recovered the expression of K10 in a concentration-dependent manner, as shown in Figure 6, without interfering with its basal expression. The expression of IVN and its impairment using IL-4 treatment were less pronounced, thus reflecting the limits related to the use of a keratinocytes monolayer. However, treatment with HVE and HT showed the partial recovery of IVN expression (Supplementary Materials, Figure S1).

**Figure 7:**
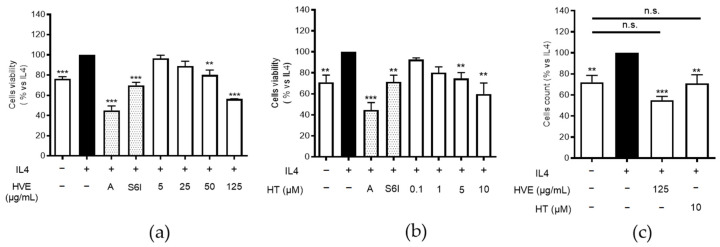
The inhibitory effect of HVE (**a**) and HT (**b**) treatment (72 h) on cell proliferation induced by IL-4 (100 ng/mL) in HaCaT cells (MTT assay). The effect of HVE (125 μg/mL) and HT (10 μM corresponding to 4.93 μg/mL) measured by the trypan blue test is also reported (**c**). The data are expressed in percentages, relative to the stimulated control, which is arbitrarily assigned a value of 100%. ** *p* < 0.01, *** *p* < 0.001 versus stimulus. HVE, *Hamamelis virginiana* bark extract; HT, hamamelitannin; reference inhibitors: A, apigenin (20 μM); S6I, STAT-6 inhibitor (50 nM). n.s., no significance.

Consequently, we decided to sustain the previous observation by assessing whether HVE and HT might also counteract the proliferative effects of IL-4. In this case, HaCaT cells were treated before reaching confluency, at the early stages of differentiation (from day 1 to day 3). In analogy with the previous data, HVE (5–125 μg/mL) impaired IL-4-induced proliferation in a concentration-dependent fashion, as shown by the MTT assay (Figure 7a). The same effect was observed for HT (0.1–10 μM) (Figure 7b), although, once again, the inhibitory concentrations were not responsible for the activity of HVE. The data were further confirmed by the direct count of living cells (the trypan blue test) after the treatment with the highest concentrations of previously tested HVE and HT (Figure 7c). The latter experiments also represented a valuable method to exclude that HVE may act through cytotoxic mechanisms, since the number of living cells was not significantly different from the control. The direct interference with cell viability, in the absence of IL-4 challenge, was already excluded by our previous MTT experiments [13].

## 3. Discussion

*Hamamelis virginiana* L. is a well-known traditional remedy used for the control of minor skin and vascular disorders in USA and Europe. Despite its long-standing application in topical products, the European HPMC assessed that the evidence of its efficacy is still too limited to define a well-established use [1]. HT and proanthocyanidins may represent the main polyphenols responsible for the biological properties [9,10,11,12], but the available clinical studies were conducted on distillates (Hamamelidis aqua), which are devoid of tannins [3,4,5]. On the other hand, the bioactivity of polar extracts in specific skin diseases, such as AD, was not investigated in pre-clinical studies until now.

We previously reported that the glycolic extract included in the present study (HVE) mainly contains HT (0.30%) and oligomeric proanthocyanidins (0.29%) [13]; however, pure HT (until 10 μM, roughly corresponding to 5 μg/mL) was not able to explain the inhibitory activity of HVE against *C. acnes* or TNF-α-induced inflammation in human keratinocytes, thus suggesting the importance of oligomeric proanthocyanidins.

In this work, we focused on biomarkers with crucial relevance for the pathogenesis of the inflammatory process occurring in AD. In line with the previous data, we observed that HT was not able to counteract IL-17C and MMP-9 release during the TNF-α challenge in HaCaT cells, diverging from HVE, which exhibited IC_50_s below 2 μg/mL. Similarly, only HVE inhibited the release of IL-6 induced by TNF-α/IFN-γ or TNF-α/IL-4 combination.

HVE also impaired TSLP release induced by the latter combination of stimuli with an IC_50_ of 4.33 μg/mL, thus suggesting potential anti-allergic properties. Surprisingly, HT displayed an inhibitory activity on the release of TSLP (IC_50_ = 2.56 μM, corresponding to 1.24 μg/mL), at concentrations slightly higher than those present in HVE.

These data suggested that HT may strictly participate in the biological activity of HVE against IL-4-dependent targets and possibly control several AD-related symptoms, such as itch and skin fragility. Of note, we also demonstrated that the IL-4/STAT6 pathway directly interacts with the NF-κB-driven transcription during TNF-α/IL-4 challenge. Since HVE impaired the NF-κB-driven transcription challenged by the cytokine milieux used in this study, we concluded that HVE may exert its effects mainly through NF-κB impairment, plausibly ascribable to proanthocyanidins. Indeed, the involvement of HT in the impairment of the NF-κB pathway was excluded, thus suggesting that HT may act through mechanisms unrelated with STAT6 and NF-κB pathways.

To better explain the inhibitory effects of HT on TSLP release and further characterize the bioactivity of HVE, we also investigated several IL-4-dependent targets.

Accordingly, we demonstrated that HVE and HT may counteract the release of CCL26 using keratinocytes, thus sustaining the possible role of HVE in controlling pruritus and eosinophilic infiltration in eczema. Moreover, HVE and HT may rebalance the proliferative status of keratinocytes during IL-4 challenge favoring the expression of K10 and epidermal differentiation. The intriguing selectivity of HT on IL-4-dependent targets demands dedicated studies which aim to identify molecular targets, considering that IL-4 is known to induce skin barrier impairment also through the activation of MAPKs [45]. Moreover, further analysis would be required to demonstrate the impact of HVE and HT on markers related to the modulation of cell turnover and skin barrier in AD models. For the purpose, our work suggests the potential investigation of *Hamamelis virginiana* L. bark in more complex models of disease, such as 3D reconstituted epidermis or in vivo models. 

In analogy with previous data, HT displayed its bioactivity at concentrations higher than those present in the extract. This evidence underlines a potential synergistic effect between HT and oligomeric proanthocyanidins, sustaining the importance of using whole extracts instead of individual compounds.

Despite the fact that hydrolysable tannins have been poorly investigated in the context of atopic eczema, our results are in line with several observations concerning the effects of other tannin-rich extracts in rodent models of atopic diseases [51]. Moreover, HT had already been suggested as a potential anti-allergic candidate for its 5-LOX inhibitory activity [12]. A very recent short communication from Choi et al. (2021) [52] demonstrated the role of hexagalloylglucose from stems and leaves of *Hamamelis virginiana* L. against PAR-2/NF-κB activation, a mechanism implied in TSLP expression. Similarly, pentagalloylglucose and tannic acid impaired IL-4/STAT6 signaling in Burkitt lymphoma cells [53]. Nevertheless, proanthocyanidins have been reported as dietary compounds with anti-allergic properties in several studies on allergic asthma [54]. Consequently, our results may stimulate further studies involving sources of tannins with potential effects against skin-inflammatory based diseases, including atopic eczema. In addition, the mechanism of action and structure–activity relationships of gallotannins in Th2-driven inflammation demand for specific investigations.

To the best of our knowledge, this is the first pharmacological validation for the use of *Hamamelis virginiana* L. extract in atopic eczema. Since HT showed bioactivity restricted to allergic markers, the present evidence gives a rational for a careful characterization of tannins in *Hamamelis virginiana* L. extracts, according to the application of specific pathological conditions. Future studies should explore the development of a formulative strategy to the context of animal models and clinical trials.

## 4. Materials and Methods

*Hamamelis virginiana* L. glycolic extract (HVE) was kindly provided by Istituto Ganassini di Ricerche Biomediche (Milan, Italy). HT was purchased from PhytoLab GmbH & Co. KG (Vestenbergsgreuth, Germany). We previously characterized HVE, which contains HT (0.3%) and galloylated proanthocyanidins (0.29%) as main polyphenolic compounds [13]. The STAT6 inhibitor (S6I) (compound AS1517499, Merck Life Science, Milan, Italy), apigenin (A) (Phytolab GmbH & Co. KG, Vestenbergsgreuth, Germany) and epigallocatechin-3-O-gallate (EGCG) (Phytolab GmbH & Co. KG) were used as reference compounds. The highest concentration of 10 μM HT (MW = 484.84 g/mol) corresponds to 4.93 μg/mL.

The vehicle of HVE was 2-methylpropane-1,3-diol, while the vehicle of HT stock solution (25 mM) was DMSO. Each vehicle was used as a control in the biological experiment, reaching concentrations always below 0.1%.

### 4.1. HaCaT Cell Culture and Differentiation

HaCaT (CVCL-0038; Cell Line Service, Eppelheim, Germany), a spontaneously immortalized human keratinocyte cell line from adult skin, was maintained at 37 °C, in a humidified atmosphere containing 5% CO_2_, in Dulbecco’s modified Eagle’s medium (DMEM; Merck Life Science, Milano, Italy), supplemented with 10% heat-inactivated fetal bovine serum (Euroclone, Pero, Italy), 100 U/mL of penicillin, 100 μg/mL of streptomycin (Gibco^TM^; Thermo Fisher Scientific, Monza, Italy), and 2 mM of L-glutamine (Thermo Fisher Scientific, Monza, Italy). For the sub-culture, cells were detached from 75 cm^2^ flasks (Primo^®^; Euroclone, Pero, Italy) every 3 days using Trypsin-EDTA 0.25% (Gibco^TM^; Thermo Fisher Scientific, Monza, Italy), counted, and placed in a new flask or seeded in plates (Falcon^®^; Corning Life Sciences, Amsterdam, The Netherlands) for the biological tests.

According to Colombo et al. and Omori-Miyake et al. [45,55], differentiated HaCaT cells were obtained by culturing cells in 24-well plates (3 × 10^4^/well) in high-calcium DMEM (1.8 mM) for 9 days, reaching over confluency. The medium was replaced every 48 h. The expressions of K10 and involucrin (IVN) were assessed as biomarkers of differentiation via immunofluorescence, as already mentioned in our previous article [44].

### 4.2. Evaluation of Cell Viability and Proliferation

The integrity of the cell morphology before and after each treatment was assessed via light microscope inspection. The cell viability was measured by the 3,4,5-dimethylthiazol-2-yl-2-5-diphenylte-trazolium bromide (MTT) method. HVE and HT showed no cytotoxicity at the highest concentration tested (250 μg/mL and 10 μM, respectively) at the end of the treatments, as already reported in our previous work [13].

The MTT test was also used as a recognized index of cell proliferation after the treatment with IL-4 (100 ng/mL) and HVE (5–125 μg/mL) or HT (0.1–10 μM) for 72 h. For this purpose, HaCaT cells were seeded in 96-well plates (10^4^ cell/well) for 24 h, and then the medium was replaced with low-FBS DMEM (1% FBS) containing the test conditions.

For a better comparison, cell proliferation was also measured using the direct cell count following the trypan blue exclusion test. In this case, cells were cultivated in 12-well plates (2 × 10^4^/well) and treated with test conditions in low-FBS DMEM, as previously described. After detachment with trypsin, trypan blue (0.4%) (Merck Life Science, Milan, Italy) was added to cell suspension (1:1) and cells were counted in cell-counting chamber slides (Becton Dickinson Italia S.p.A., Milano, Italy). S6I (25 nM) and apigenin (5 μM) were used as reference compounds.

### 4.3. Evaluation of Cytokeratin 10 (K10) and Involucrin (IVN) Expression via Immunofluorescence

HaCaT cells were cultured on glass coverslips in 24-well plates (3 × 10^4^/well) for 9 days. Then, cells were treated for 24 h with IL-4 (100 ng/mL) and HVE (5 and 50 μg/mL) or HT (1 and 5 μM). Cells were fixed via incubation with formaldehyde (4%) for 15 min, and then they were washed 3 times with PBS 1X. The blocking-permeabilizing solution was added (BSA 5%, Triton-X 0.3%, in PBS 1X) for 1 h, and then immunostaining with anti-K10 (3C2F5) (1 μg/mL) and anti-IVN (LHK1) (2 μg/mL) mouse antibodies (Novus biologicals, Milan, Italy) was performed at 4 °C overnight. The day after, the AlexaFluor^®^ 488-conjugated anti-mouse antibody (Cell Signaling, MA, USA) was added for 1 h, and then cells were washed 3 times and mounted on glass slides with ProLong Gold Antifade DAPI (Cell Signaling, MA, USA). The fluorescent images were acquired using confocal microscopy (LSM 900, Zeiss, Oberkochen, Germany). 

### 4.4. Measurement of Cytokine Release with the ELISA Assay

HaCaT cells were cultured in 24 well plates (3 × 10^4^/well) for 48 h (undifferentiated) or 9 days (differentiated), and then cytokine release was evaluated after 24 h of stimulation and treatment with HVE or HT. The stimulatory conditions are briefly stated below:

- IL-17C and MMP-9 release was stimulated with TNF-α (10 ng/mL) in undifferentiated HaCaT cells;
- TSLP and IL-6 release was stimulated with TNF-α (10 ng/mL)/IL-4 (100 ng/mL) combination in differentiated HaCaT cells, and IL-6 was also evaluated after TNF-α (10 ng/mL)/IFN-γ (5 ng/mL) stimulation in undifferentiated HaCaT cells, according to our previously published time-course experiments [44];
- CCL26 release was stimulated with IL-4 (100 ng/mL) in differentiated HaCaT cells.

The release of the above-mentioned mediators was evaluated with the enzyme-linked immunosorbent assay (ELISA) using culture media. The release of MMP-9 was measured with the human MMP-9 ELISA kit (RayBiotech Life, Inc. 3607 Parkway Lane Suite 200, GA, USA), according to manufacturer’s instructions. Human TSLP, IL-6, and CCL26 ELISA development ABTS kits were purchased from PeproTech (PeproTech, London, UK). Coating anti-human IL-17C antibody and biotinylated anti-IL-17C were purchased from Novus (Novus biologicals, Bio-Techne s.r.l., Milan, Italy). 

Corning 96-well EIA/RIA plates (Merck Life Science, Milano, Italy) were coated overnight at room temperature with the capture antibodies. The amounts of TSLP, IL-6, CCL26, and IL-17C in the samples were detected by measuring the absorbance resulting from the colorimetric reaction between the horseradish peroxidase enzyme and the 2,20-azino-bis (3-ethylbenzothiazoline-6-sulfonic acid) (ABTS) substrate (Merck Life Science, Milan, Italy), according to the manufacturer instructions. The signal was read using a spectrophotometer (VICTOR X3; PerkinElmer, Milano, Italy) at 405 nm. The results (mean ± SEM of at least three experiments) were expressed as a percentage relative to the stimulated control, which was arbitrarily assigned to a value of 100%.

S6I (50 nM) and apigenin (20 μM), considered as reference inhibitors of the IL-4/STAT-6 pathway [28,29], were used as inhibitors of IL-4-induced type 2 mediators (TSLP, IL-6, and CCL26). EGCG (20 μM) was used as a reference inhibitor of MMP-9 and IL-17C release [56,57].

### 4.5. Measurement of the NF-κB-Driven Transcription

HaCaT cells were cultured in 24-well plates for 48 h, and then transiently transfected with a reporter plasmid responsive to NF-κB (250 ng per well), containing the luciferase gene under the control of the E-selectin promoter characterized by three κB-responsive elements. Lipofectamine^®^ 3000 transfection reagent (Invitrogen^®^, Waltham, MA, USA; Thermo Fisher Scientific, Monza, Italy) was used for the transfection assays. The plasmid was a gift from Dr. N. Marx (Department of Internal Medicine-Cardiology, University of Ulm; Ulm, Germany). A few day later, the cells were treated with TNF-α/IFN-γ or TNF-α/IL-4, in addition to HVE (5–125 μg/mL) or HT (1–10 μM) for 6 h. At the end of the treatment, the amount of luciferase produced into the cells was assessed using the Britelite^TM^ Plus reagent (Perkin Elmer, Milano, Italy), according to the manufacturer’s instructions. The luminescence deriving from the reaction between luciferase and luciferin was measured with a VICTOR X3 multilabel plate reader (Perkin Elmer, Milano, Italy). The results (mean ± SEM of at least three experiments) were expressed as a percentage relative to the stimulated control, which was arbitrarily assigned to a value of 100%. S6I (50 nM) and apigenin (20 μM) were used as reference inhibitors of TNF-α/IL-4 stimulation, while EGCG (20 μM) was used as a reference inhibitor of TNF-α/IFN-γ stimulation.

### 4.6. Statistical Analysis

All data were expressed as the mean ± SD of at least three independent experiments. The quantitative assays were analyzed using unpaired one-way analysis of variance (ANOVA), followed by the Bonferroni post-hoc test. Statistical analyses were performed using GraphPad Prism 8.0 software (GraphPad Software Inc., San Diego, CA, USA). Values of *p* < 0.05 were considered statistically significant. 

## Data Availability

The data presented in this study are available upon request from the corresponding author.

## Supplementary Materials

The following supporting information can be downloaded at: https://www.mdpi.com/article/10.3390/ijms23169279/s1.
