# Supplementary material for: Unveiling the Ability of Witch Hazel (Hamamelis virginiana L.) Bark Extract to Impair Keratinocyte Inflammatory Cascade Typical of Atopic Eczema
Source: Int J Mol Sci. 2022 Aug 17;23(16):9279. doi: 10.3390/ijms23169279 (PMC9408886; doi:10.3390/ijms23169279)

Supplementary material

Figure S1. Immunofluorescence images reporting the effect of HVE and HT treatment (24h) on the impairment of involucrin (IVN) expression induced by IL-4 (100 ng/mL) in differentiated HaCaT cells. HVE, *Hamamelis virginiana* bark extract; HT, hamamelitannin. Green, IVN staining; blue, DAPI (nuclei) staining.

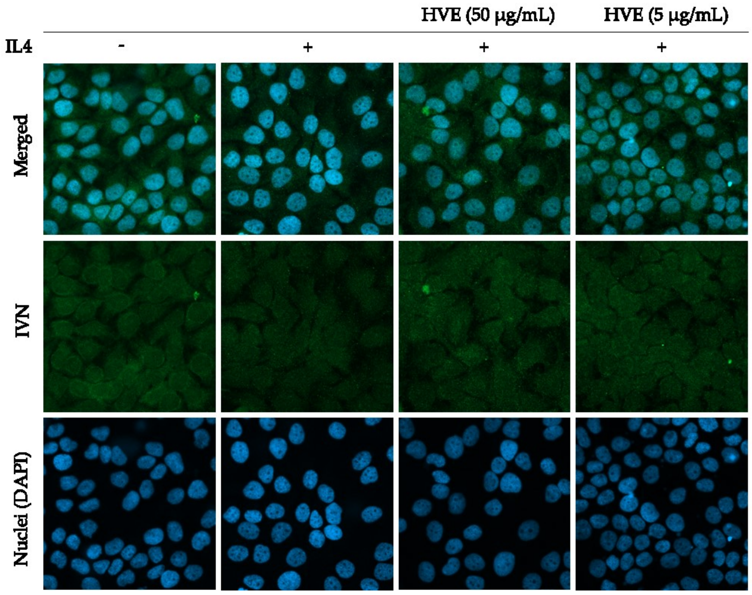

Supplement: Supplementary file 1 [file ijms-23-09279-s001.zip › ijms-1837684-supplementary.pdf]
